# Comparative Pathology and Ecological Implications of Two Myxosporean Parasites in Native Australian Frogs and the Invasive Cane Toad

**DOI:** 10.1371/journal.pone.0043780

**Published:** 2012-10-03

**Authors:** Ashlie Hartigan, Navneet K. Dhand, Karrie Rose, Jan Šlapeta, David N. Phalen

**Affiliations:** 1 Faculty of Veterinary Science, University of Sydney, New South Wales, Australia; 2 Australian Registry of Wildlife Health, Taronga Conservation Society Australia, Mosman, New South Wales, Australia; Australian Wildlife Conservancy, Australia

## Abstract

Myxosporean parasites *Cystodiscus axonis* and *C. australis* are pathogens of native and exotic Australian frog species. The pathology and ecological outcomes of infection with these parasites were investigated in this study. Gliosis was correlated to *Cystodiscus axonis* plasmodia in the brains of (9/60) tadpoles and (3/9) adult endangered Green and golden bell frogs using ordinal regression. Severe host reactions to *C. axonis* (haemorrhage, necrosis, and vasulitis) were observed in the brains of threatened Southern bell frogs (8/8), critically endangered Booroolong frogs (15/44) and Yellow spotted bell frogs (3/3). Severe brain lesions were associated with behavioural changes, neurological dysfunction, and spontaneous death. Both *C. axonis* and *C. australis* develop in the bile ducts of tadpoles, the plasmodia were significantly associated with biliary hyperplasia, inflammation and the loss of hepatocytes in (34/72) Green and golden bell frog tadpoles using ordinal regression. These lesions were so severe that in some cases 70% of the total liver was diseased. Normal liver function in tadpoles is necessary for metamorphosis, metabolism, and immune function. We postulate that this extensive liver damage would have significant host health impacts. Severe hepatic myxosporidiosis was more prevalent in tadpoles examined in autumn and winter (overwintered), suggestive of delayed metamorphosis in infected tadpoles, which would have serious flow-on effects in small populations. We compared the sensitivity of histopathology and species-specific PCR in the detection of *C. australis* and *C. axonis*. PCR was determined to be the most sensitive method (detection limit 1 myxospore equivalent of ribosomal DNA). Histology, however, had the advantage of assessing the impact of the parasite on the host. It was concluded that these parasites have the potential for significant ecological impacts, because of their high prevalence of infection and their ability to cause disease in some frogs.

## Introduction

Myxosporea are two host metazoan parasites, closely related to jellyfish (Cnidarian) [1]–[3] with over 2, 100 species of myxosporean parasites identified to date [4]. Intermediate hosts are primarily aquatic vertebrates including fish, reptiles, and amphibians. Recently however, myxosporea have been identified in shrews (*Sorex araneus*) [5], [6], a mole (*Talpa europaea*) [7] and several species of waterfowl [8]. Myxosporean parasites can be either species specific or can infect a range of closely related species. Known definitive hosts for Myxosporea are annelids (oligochaetes and polychaetes), however only 34 life cycles have been identified so far [9]. Myxosporea can develop in different tissues of their intermediate hosts, including gills, muscle, intestine, urinary tract, gonad, gallbladder, liver, bone and the central nervous system [9]–[11]. The impact of infection on the intermediate host can range from minimal to severe. *Myxobolus cerebralis*, a parasite of salmonid fish, has caused morbidity and mortality in captive raised and wild fish wherever the parasite has been introduced to susceptible species [12]–[16]. *Chloromyxum truttae*, in contrast, is a common parasite in the same salmonid range as *M. cerebralis* and yet causes no host reaction in wild fish [17], [18].

Two myxosporean species, *Cystodiscus axonis* and *Cystodiscus australis*, have recently been characterized in Australian frogs [19], [20]. Both *Cystodiscus axonis* and *C. australis* have presporogonic stages in bile ducts that are followed by the development of spore producing plasmodia in the gallbladder. *Cystodiscus axonis* is unique in that it has a presporogonic stage that develops in the central nervous system. Lesions in the central nervous system often accompany infection with *C. axonis* and liver lesions are found with infection by both of these parasites in some species of frogs, but not others. The origin of these parasites is not known, but both appear to have emerged in Australian frogs in the mid 1960's [21]. One or both of these parasites have now been identified in 7 Australian frogs species (*Litoria aurea, Litoria peronii, Litoria raniformis, Litoria castanea, Litoria booroolongensis, Limnodynastes peronii, Rhinella marina*), representing 3 (Hylidae, Myobatrachidae, Bufonidae) out of 5 frog families in Australia [19], [20], [22]. Importantly, infections and associated lesions have been found in 4 endangered species (*L. aurea, L. raniformis*, *L. booroolongensis* and *L. castanea*). The populations of each endangered frog in which these parasites have been identified began declining from the 1970's and 80's [23]–[27] which is approximately the time that these *Cystodiscus* species first emerged [21].

Infectious diseases are known to be important drivers of population declines and extinctions in amphibians and other species of animals. Disease causing organisms however, are often found in healthy populations of animals and, proving that organisms are causing significant disease and are having deleterious impacts on a population may be difficult [28]. This process is especially difficult when organisms cannot be cultured and controlled infection trials cannot be undertaken.

It is the purpose of this study to document and statistically evaluate *C. axonis* and *C. australis* as agents of significant disease and their potential to impact the survivability of both tadpoles and adult frogs. We compared the sensitivity and specificity of different techniques (histology and PCR) that can be used for diagnosis in individual animals and for population surveillance. Lastly, we provide evidence that differing host responses may play a role in the outcome of infection in these two parasites and show how the host-pathogen-environment interactions may have important ecological implications.

## Methods

### Ethics Statement

Green and golden bell frogs, Striped marsh frogs, Peron's tree frogs and Cane toads were collected under Scientific license numbers 12686 and 12969 (Office of Environment and Heritage, Australia). Animals were euthanized with AQUI-S (AQUI-S, New Zealand) [29] according to University of Sydney Animal Ethics Licenses (N00/9-2008/3/4855 and N00/9-2009/3/5134). Dead Yellow spotted bell frogs, Southern bell frogs and Booroolong frogs were examined through the opportunistic sample collection program under the auspices of the Taronga Conservation Society Australia Animal Ethics Committee.

### Specimens

Green and golden bell frog (*Litoria aurea*) tadpoles (n = 97), metamorphs (n = 1) and adults (n = 11); Striped marsh frog (*Limnodynastes peronii*) tadpoles (n = 61) and adults (n = 27) and Peron's tree frog (*Litoria peronii*) tadpoles (n = 18), metamorphs (n = 1) and adults (n = 7) were collected from a semi-captive breeding colony for Green and golden bell frogs in Riverstone, NSW (−33.679, 150.860) from May 2008 to December 2010 (Table 1). Green and golden bell frogs were bred at this facility in multiple man-made pools. Adult Green and golden bell frogs, Striped marsh frogs and Peron's tree frogs spawned and moved freely in and out of the pools. Both *Cystodiscus axonis* and *C. australis* have previously been shown to be enzootic to this breeding facility [19], [20].

**Table 1:** Summary of species, age and tissue samples examined.

Species	Age	Total number examined	Brains examined	Brain positive	Range in parasite load (brain)	Livers examined	Liver positive	Range in hepatitic myxosporidiosis severity	Gallbladder myxospores positive	Fibrosis (F)/Amyloid-like tissue in liver (A)
Green and golden bell frog	Tadpoles	97	60	9 (15.0%)	None = 51	72	34	None = 15	0	0
(*Litoria aurea*)					Mild = 6			Mild = 16		
					Moderate = 3			Moderate = 28		
								Severe = 13		
	Adults	12	9	3 (33.3%)	None = 6	12	1*	None = 12	1	1 – F + A
					Mild = 1					
					Moderate = 2					
Southern bell frog	Adults	8	8	8 (100%)	Mild = 2	8	0	None = 7	7	8 – F + A
(*Litoria raniformis*)					Moderate = 3					
					Severe = 2					
Booroolong frog	Adults	44	44	15 (34.1%)	Mild = 9	38	3	None = 38	n.a.	3 – F + A
(*Litoria booroolongensis*)					Moderate = 6					
Yellow spotted bell frog	Adults	3	3	3 (100%)	Severe = 3	3	1*	None = 2	n.a.	2 – F + A
(*Litoria castanea*)								Mild = 1		
Peron's tree frog	Tadpoles	18	11	7 (63.6%)	Mild = 7	14	12	None = 1	0	0
(*Litoria peronii*)								Mild = 4		
								Moderate = 7		
								Severe = 2		
	Adults	8	6	3 (50.0%)	Moderate = 2	8	1*		5	4 – F + A, 1 – F only
					Severe = 1					
Striped marsh frog	Tadpoles	61	42	0	None = 42	38	10	None = 29	0	0
(*Limnodynastes peronii*)								Mild = 9		
	Adults	27	17	2 (11.7%)	Moderate = 1	25	0	None = 24	20	12 – F only
					Severe = 1			Mild = 1		
Cane toad	Tadpoles	14	12	0	None = 12	14	0	None = 14	0	0
(*Rhinella marina*)	Adults	27	26	2 (7.7%)	None = 24	27	0	None = 27	9	6 – F only
					Mild = 1					
					Severe = 1					

Booroolong (*Litoria booroolongensis*) frogs (n = 44) were collected from two locations, Maragle Creek (n = 25) and Abercrombie (n = 19) in NSW, Australia between 2007 and 2010. These animals were kept at the Taronga Zoo, Sydney, Australia for variable lengths of time and were either found dead or were euthanased as part of a disease screening program or due to some abnormal behaviour (e.g. leg dragging). Eight Southern bell frogs (*Litoria raniformis*) were wild caught in southern NSW, Australia in 2006 and kept in captivity until 2009 when they were euthanased due to lethargy and lack of breeding. Fourteen Yellow spotted bell frogs (*Litoria castanea*) were wild caught from southern NSW in 2010 and held in a captive breeding colony. Three frogs (1 sub-adult and 2 adults) died spontaneously and were submitted for necropsy.

Cane toad (*Rhinella marina*) tadpoles (n = 14) and adults (n = 31) were collected from areas around Lismore, NSW (−28.813, 153.279) in February 2009 and 2010, at the southern end of their distribution in eastern Australia.

### Tissue processing

Tadpoles were bisected along the mid line. One half was fixed in 10% buffered formalin, paraffin embedded, and 5 µm sagittal sections of entire tadpoles were stained with haematoxylin and eosin. Brain, liver, spinal cord, kidney, spleen, and reproductive organs from adult frogs were individually fixed, and similarly sectioned and stained. A section through the cervical, thoracic and lumbar spinal cord was examined for each adult frog.

Liver sections from adult frogs (n = 3) were stained with Congo red to determine the presence of amyloidosis. For PCR analysis, brain, brainstem or liver were separately removed from the unfixed half of the tadpoles and adult frogs, or unfixed half of the brain stem and a representative portion of the liver of adult frogs. Individual tissues were stored at −80°C until processing.

Many of the tadpoles were very small. In these cases, it was not always possible to get tissue for both histopathology and the PCR assay and therefore, tissues were only examined by histopathology.

### Evaluation of disease

#### Behavioural observations of captive frogs

Southern bell frogs, Booroolong frogs and Yellow spotted bell frogs were maintained at Taronga Zoo, Sydney, Australia. All animals were subject to zoo staff observations; behavioural abnormalities were noted in individual pathology and diagnostic reports within the Australian Registry of Wildlife Health.

#### Assessment of lesions

A summary of the species and tissues examined to build a syndrome description is provided within Table 1.

##### Association of C. axonis infection with microscopic lesions of the central nervous system of the Green and golden bell frog

Gliosis was defined as increased numbers of glial cells occurring focally or more diffusely within a section of the brain or spinal cord. *Cystodiscus axonis* infection and the presence of gliosis and other lesions in sagittal sections of the brain and spinal cord was recorded from 9 Green and golden bell frog tadpoles. Sagittal sections through the brain and 3 transverse sections through the spinal cord were also examined in 6 Green and golden bell frog adults. When an infected tadpole or frog was found, the severity of infection was assessed using the area of the central nervous system where the parasites were in highest concentration. Once this area was identified, parasite load was graded into three classes depending on the number of organisms that could be seen on 10X magnification: mild (1–5 plasmodia), moderate (6–10 plasmodia) and severe (more than 10 plasmodia). The entire brain and spinal cord sections were examined for evidence of host response to the parasite. Gliosis was recorded as either present or absent and when applicable, lesions such as perivascular lymphoid cell cuffing, malacia or haemorrhage were recorded.

##### Association of infection with Cystodiscus species and microscopic lesions in the liver of Green and golden bell frog

To examine the possible relationship between *Cystodiscus* species infection and microscopic liver disease, 72 tadpoles, 1 metamorph (here after included in the analysis for adults), and 11 adult Green and golden bell frogs were examined. Each liver section was examined for the presence or absence of plasmodial stages within the bile ducts, biliary hyperplasia, lymphoplasmacytic inflammation, loss of hepatocytes and biliary fibrosis. Bile duct plasmodia were recorded as either present or absent. The severity of the liver lesions were classified as mild (10–30% of liver affected), moderate (30–60% of the liver affected) and severe (60% or more of the liver affected) when a representative section of the liver was viewed under 10X magnification. It has been previously shown that *C. axonis* and *C. australis* are morphologically indistinguishable in liver sections and both infections can occur simultaneously [20]. In this study, it was not possible to determine which parasites were present in the liver. Thus the observed lesions could be associated with *C. axonis*, *C. australis* or a combined infection.

##### Statistical association of microscopic lesions with Cystodiscus species infection in Green and golden bell tadpoles and frogs

Associations were initially investigated by creating contingency tables of the presence of infection with presence of lesions and lesion severity. Univariable binary logistic regression analyses were then conducted to determine association between infection, age and season and the binary outcome variables – presence or absence of lesions in the brain (Supplementary Table 1A) and liver (Table S1D) for the tadpoles and adult Green and golden bell frog. Similar binary logistic regression models were constructed to evaluate the association of age and season with presence or absence of infection in brain and liver. For ordinal outcome variables (e.g. severity, graded with none, mild, moderate and severe) ordinal logistic regression analyses were conducted to determine their association with infection, age and season. Assumption of proportional odds for ordinal models was evaluated using Score test. Multivariable logistic regression analyses were then conducted (binary or ordinal, as appropriate) to evaluate the association of infection, age and season after adjusting for each other. First order interaction terms were finally tested by adding to the multivariable model and retained if significant. Fisher's exact test was used to evaluate associations where logistic regression model did not converge because some of the cell frequencies were zero.

SAS statistical software (release 9.3, 2002–10, SAS Institute Inc., Cary, NC, USA) and UniLogistic macro (Dhand, 2010) were used for all analyses. Odds ratios are reported with 95% confidence intervals (CI) and 2-sided *p*-values.

Prevalence data for *Cystodiscus* plasmodia in Green and golden bell frog tadpole livers and *C. axonis* in brains was compared to other species of tadpoles (Striped marsh and Peron's tree frog tadpoles) that were collected from the same location (Riverstone, NSW) in the same season. The overall differences in prevalence were evaluated with χ^2^ tests or Fisher's exact test if the cell frequencies were low. If the overall differences were significant, pair-wise comparisons were made using Z-test for comparing proportions or Fisher's exact test, as appropriate.

### Assessment of the impact of season on the presence and severity of lesions in the Green and golden bell frog tadpoles and adult frogs

Three histological parameters were mapped in Green and golden bell frogs across seasons between May 2008 and December 2010. Seasons were defined as autumn (March, April, May), winter (June, July, August), spring (September, October, November) and summer (December, January, February). The presence or absence of *Cystodiscus* spp. infection of the liver and the degree of severity of associated lesions in the liver seasonally were examined in tadpoles only (n = 72). Similarly, the presence or absence of *Cystodiscus axonis* infection in the brain was examined in tadpoles only (n = 60) (Table 1).

Due to the small amount of tissue available in a tadpole, not all sagittal sections contained both brain and liver. Therefore the numbers of animals that were included in the liver and brain seasonality study were not always the same (Table 1 and 2). Association between the lesion of interest and season was analysed with ordinal logistic regression and when necessary (brain plasmodia) Fisher's exact test in SAS (Table S1A and D).

**Table 2:** Seasonal prevalence of *Cystodiscus axonis* plasmodia, *Cystodiscus* spp. liver plasmodia and hepatic myxosporidiosis in Green and golden bell tadpoles.

A					
Brain plasmodia	Spring	Summer	Autumn	Winter	n
Plasmodia absent	6 (75.0%)	14 (82.3%)	7 (63.6%)	24 (100%)	51
Plasmodia present	2 (25.0%)	3 (17.6%)	4 (36.4%)	0 (0%)	9
Total	8	17	11	24	60

B					
Liver plasmodia	Spring	Summer	Autumn	Winter	n
Plasmodia absent	7 (77.8%)	8 (57.1%)	7 (35.0%)	16 (55.1%)	38
Plasmodia present	2 (22.2%)	6 (42.9%)	13 (65.0%)	13 (44.8%)	34
Total	9	14	20	29	72

C					
Severity of hepatic myxosporidiosis	Spring	Summer	Autumn	Winter	n
None	5 (55.6%)	6 (42.9%)	3 (15.0%)	3 (10.3%)	17
Mild	3 (33.3%)	4 (28.6%)	9 (45.0%)	10 (34.5%)	26
Moderate	1 (11.1%)	2 (14.3%)	4 (20.0%)	10 (34.5%)	17
Severe	0	2 (14.3%)	4 (20.0%)	6 (20.7%)	12
Total	9	14	20	29	72

### Comparison of histology and multiplex PCR to detect *Cystodiscus* species infection in the brain and liver

A minimum of twelve serial sections were obtained for a sub-sample of the frog specimens (not all samples qualified due to small tissue size available). Depending on the size of the tissue, the number of 5 µm sections ranged from 12 to 34 in liver (n = 17) and 12 to 48 in brain (n = 15). Each section was screened individually and presence of *Cystodiscus* spp. was recorded. The liver specimens were all from tadpoles of the Green and golden bell frogs. The brain specimens were from the Green and golden bell frogs (tadpoles, n = 5; adults, n = 1), Peron's tree frog (adult, n = 1) and Striped marsh frog (tadpoles, n = 5; adults, n = 4).

For a subset of specimens for which a tissue sample was available in −20°C or −80°C, we isolated DNA and used a multiplex species specific PCR according to a previously published protocol [20]. The liver specimens were from Green and golden bell frogs (tadpoles, n = 11) and Striped marsh frog (tadpoles, n = 8). The brain specimens were from the Green and golden bell frogs (tadpoles, n = 2), Peron's tree frog (adult, n = 1) and Striped marsh frog (tadpoles, n = 4; adults, n = 4).

The sensitivity of single histological section to demonstrate presence of the parasites in tissue compared with serial section and PCR and vice versa was calculated using a standard equation (sensitivity of a test  =  # of true positives/# of true positives + # of false negatives). The true positives were considered those returning positive outcomes in histology or PCR.

To determine the detection limit of the multiplex species specific PCR [20] we tested samples with a known number of *C. axonis* and *C. australis* myxospores (in PBS counted by haemocytometer). The DNA isolation and PCR reactions were performed according to Hartigan et al. [19], all DNA samples were eluted in 50 µl buffer. Extracted DNA was diluted 10-fold to the equivalent approximately <0.01 myxospore per µl (calculated myxospores in extraction/eluted volume). PCR reactions (25 µl) contained 1 µl of template DNA and were run in triplicate, with positive and negative control in each PCR batch. Results were visualised on a 2% agarose gel stained with GelRed (Biotium, Australia).

## Results

### Distribution of *Cystodiscus axonis* in nervous system and association with gliosis in Green and golden bell frogs and tadpoles

**Figure 1:**
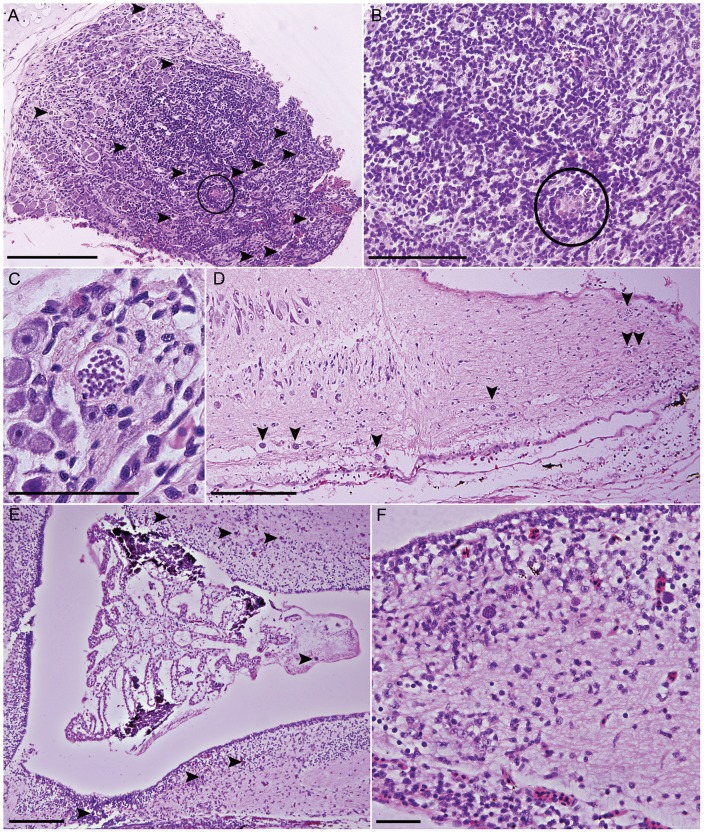
*Cystodiscus axonis* lesions in brains of Green and golden bell frogs and tadpoles. A. Root nerve of a Green and golden bell tadpole showing severe inflammation, loss of nerve ganglia and numerous *C. axonis* plasmodia (arrowheads), scale 200 µm; B. Higher magnification (20X view) of root nerve (A), infiltrate has completely replaced nerve cells, scattered foci of necrosis are seen throughout the nerve (circle), scale 100 µm; C. Higher magnification view of *C. axonis* plasmodia within the same nerve root (A) (40X), scale 50 µm; D. Posterior end of Green and golden bell metamorph spinal cord showing moderate glial activity in response to moderate plasmodia load (arrowheads indicate plasmodia). This animal had motor dysfunction possibly due to the high load of plasmodia in its spinal cord and dorsal nerve roots, scale 200 µm; E. Moderate load of plasmodia (arrows) in diencephalon and mesencephalon of tadpole, scale 200 µm; F. Higher magnification (20X) view of plasmodia in diencephalon of tadpole (E) showing increased glial activity in response to plasmodia presence, scale 40 µm.

*Cystodiscus axonis* produce 5–25 µm multinucleated non-sporogonic producing plasmodia within axons [19], [20] (Fig. 1). Plasmodia were found in 15% (9/60) of the Green and golden bell frog tadpoles and 33% (3/9) of adults (Table 1). Plasmodia were not consistently seen in all portions of the brain in all specimens. Organisms were rarely found the diencephalon (8%, 1/12) at the level of the optic lobe, but were more common caudally in the mesencephalon (34%, 4/12) and the rhombencephalon (34%, 4/12) (Fig. 1E). Plasmodia were seen in one specimen in a cranial nerve as it left the brain and in a section of the trigeminal nerve as it passed through muscle. Plasmodia were never seen in the prosencephalon, they were typically more abundant in the spinal cord (67%, 8/12) and nerve roots (50%, 6/12) (Fig. 1A, B) and if found in one nerve root, were generally found in multiple other nerve roots. In one frog plasmodia were found in a peripheral nerve. There was no association between age (adult or tadpole) and the presence of plasmodia (p = 0.21) (Table S1A).

Inflammatory changes were observed in 83% of infected animals (10/12); changes were characterised by increased numbers of glial cells (gliosis) and the response varied between focal to multifocal and locally extensive in different intensities (Fig. 1). The gliosis was often in close proximity to plasmodia (Fig. 1), but in some circumstances gliosis could be seen in areas of the nervous system where there were no plasmodia. Gliosis was observed in the brain (Fig. 1E, F), spinal cord (Fig. 1C), cranial nerves, spinal nerves and dorsal root ganglia (Fig. 1A–C). Gliosis was often the most severe in nerve roots and the dorsal root ganglia. A mild to moderate degree of malacia occurred concurrently in the brain, spinal cord and ganglia (Fig. 1A, B) of some specimens, but it was rare. Haemorrhage was not seen in the Green and golden bell frog specimens.

Gliosis is a nonspecific reaction and can be caused by multiple infectious agents [30], it was significantly associated with the presence of *C. axonis* plasmodia (*p*<0.001, odds ratio  = 42.5) (Table S1B). Gliosis was found in 78% of the (7/9) of infected tadpoles and 100% of the (3/3) of infected adults. Gliosis was either seen as a diffuse and extensive (40%, 4/10) or multifocal and locally extensive (50%, 5/10) with the exception of one animal with a single focus of gliosis (10%, 1/10). Glial activity was found in brains of 10% (6/60) tadpoles, for which plasmodia were not identified. Moderate parasite loads (6–10 cysts per view) were significantly associated with the presence of gliosis (43%, 5/12, p≤0.001, Table S1B).

### Absence of *Cystodiscus axonis* plasmodia in brains of overwintered Green and golden bell frog tadpoles

*Cystodiscus axonis* plasmodia were found in the nervous system of Green and golden bell frog tadpoles in the spring, summer and autumn (Table 2A). The prevalence of *C. axonis* infection in the nervous system was lowest in the summer (18%, 3/17), then spring (25%, 2/8) and was significantly higher (36%, 4/11) in the autumn (Table S1A, p = 0.003). No tadpoles with brain stages of *C. axonis* were identified in winter (n = 25) (Table 2A).

### Severe host reactions and increased prevalence of *Cystodiscus axonis* in threatened frog species compared to common frog species

#### Tadpoles

Overall chi-sq analysis for brain plasmodia presence in tadpoles was found to be significant (p = <0.001). Prevalence of infection in the Peron's tree frog tadpoles (64%, 7/11) was significantly higher than that observed in the Green and golden bell frog tadpoles (Table 1 and Table S1B, χ^2^ p = 0.0004). None of the Striped marsh frog tadpoles were found to be infected (0% 0/42) (Table 1 and Table S1B, 0% χ^2^ p = 0.008). The numbers of organisms found in the brains of the Peron's tree frog tadpoles was low (100% mild infection load) and while gliosis was seen in these tadpoles, the severity was less than that seen in infected Green and golden bell frog tadpoles. Haemorrhage within the nervous system was not present in any of the Peron's tree frog tadpoles.

#### Adult frogs

The prevalence of *C. axonis* in other species of frogs varied significantly. The highest was the Southern bell frogs (100%, 8/8) and Yellow spotted bell frogs (100%, 3/3). Moderate prevalences were seen in the Peron's tree frogs (50%, 3/6) and the Booroolong frogs (34.1%, 15/44). Low prevalences were observed in Striped marsh frogs (12%, 2/17) and Cane toads (8%, 2/26) (Table 1).

**Figure 2:**
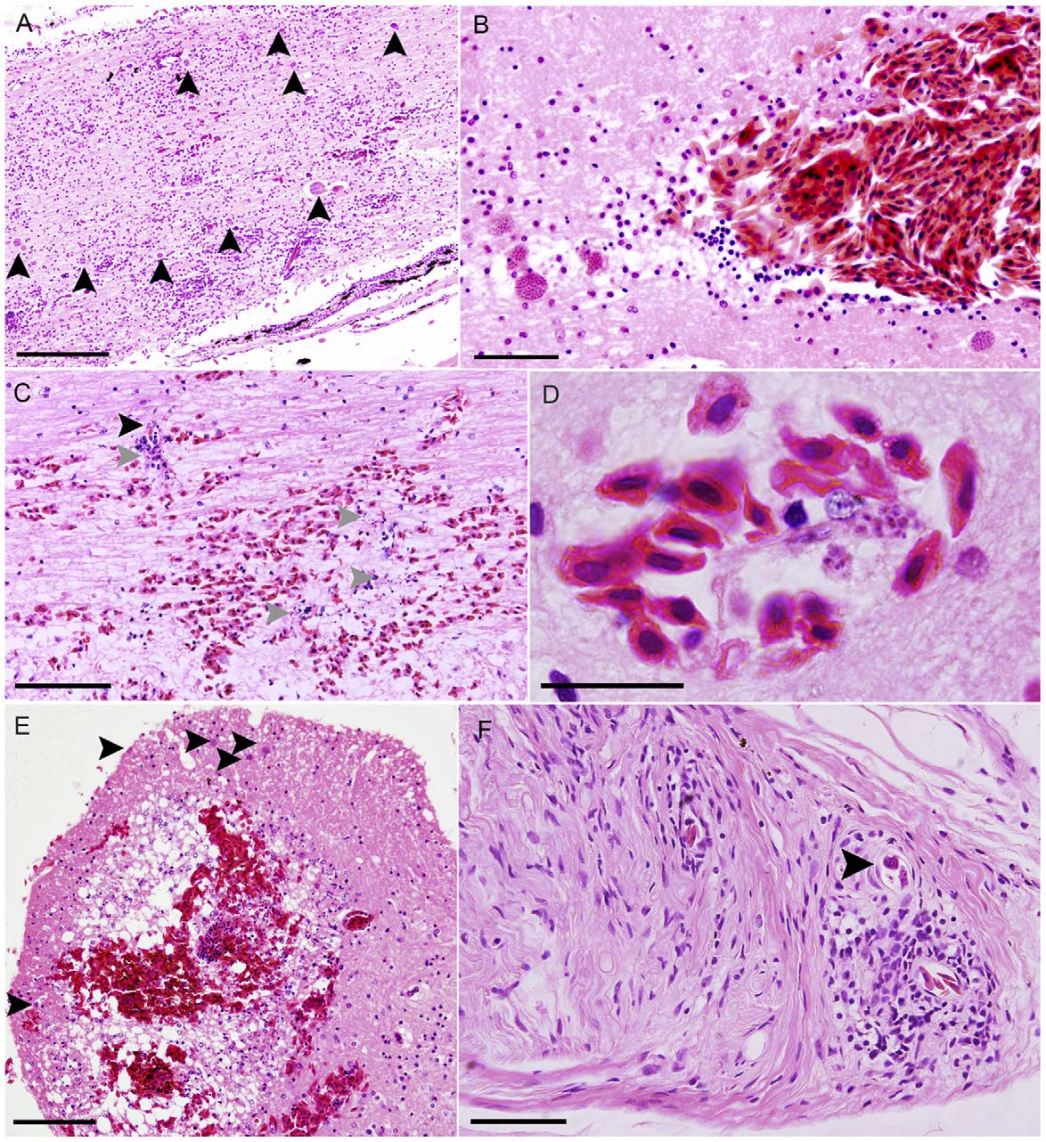
Severe reactions to *Cystodiscus axonis* plasmodia in other species of Australian frogs. A. Spinal cord of Southern bell frog showing multifocal and locally extensive gliosis in reaction to moderate load of *C. axonis* plasmodia (arrows), individual was noted to be lethargic and inappetent, scale 200 µm; B. Spinal cord of Southern bell frog showing congestion and severe haemorrhage in association with high numbers of *C. axonis* plasmodia, individual was found dead, scale 50 µm; C. Diffuse haemorrhage, multifocal melacia (grey arrowheads) and gliosis and *C. axonis* plasmodia (black arrowheads) in spinal cord of Yellow spotted bell frog, scale 100 µm; D. *C. axonis* plasmodia associated with blood vessel epithelial cell in Yellow spotted bell frog spinal cord, scale 20 µm; E. Cross section of Booroolong frog infected with *C. axonis*, showing severe haemorrhage, extensive multifocal gliosis and spongiform change, scale 100 µm; F. Nerve root of Striped marsh frog showing mild focal gliosis in association with *C. axonis* plasmodia (arrow), scale 50 µm.

Varying degrees of gliosis were found within the spinal cord (Fig. 2A–D), brain (Fig. 2E) and nerve root (Fig. 2F) sections of infected adults. Multifocal to locally extensive haemorrhage was seen in the adult Yellow spotted bell frogs (100%, 3/3) and Southern bell frogs (38%, 3/8). Haemorrhagic lesions were most severe and widespread in the Yellow spotted bell frogs (Fig. 2C and D). Vasculitis characterized by perivascular lymphoid cell cuffing, necrosis of the vascular endothelium and hylanization of the vessel walls was found in both Yellow spotted bell frogs and one Booroolong frog. Some degree of necrosis and malacia (Fig. 2C and E) was present in Yellow spotted bell frogs, Southern bell frogs and Booroolong frogs.

### Behavioural abnormalities in adult frogs with *Cystodiscus axonis*

A Green and golden bell frog metamorph was captured because it could not use its back legs. This animal had many organisms and associated gliosis in the dorsal root ganglia and spinal nerves (Fig. 1D). Three of the infected Booroolong frogs (20%, 3/15) had a history of neurologic disease. The animals were dragging their back legs and one could not right itself or react to simple stimuli. Seven of the fifteen (46.7%) Booroolong frogs were found dead. Seven of the (87.5%, 7/8) Southern bell frogs were reported to be lethargic and unresponsive to stimuli prior to euthanasia, the eighth animal was found dead. All of Yellow spotted bell frogs (3/3) died spontaneously with no observed behavioural abnormalities.

### Hepatic myxosporidiosis in tadpoles characterised by the association between biliary hyperplasia, inflammation, loss of hepatocytes and the presence of *Cystodiscus* spp. plasmodia

**Figure 3:**
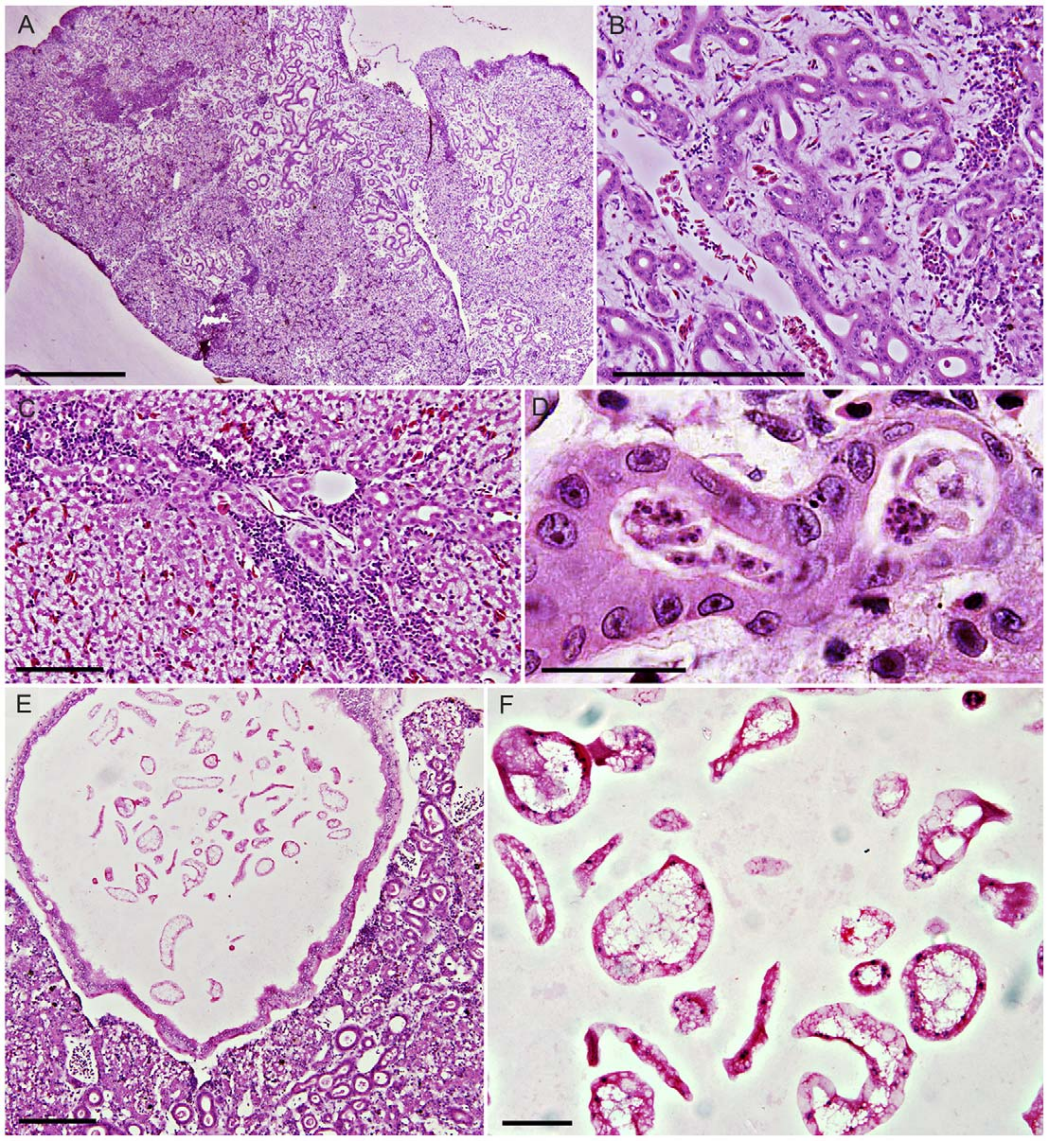
Myxosporiodiosis and developing *Cystodiscus* spp. plasmodia in tadpoles. A. Coalescing extensive biliary lesions in Green and golden bell tadpole, scale 500 µm; B. Green and golden bell tadpole showing loss of hepatocytes surrounding hyperactive biliary tree and lymphoplasmacytic inflammation, moderate grade of hepatic myxosporidiosis, scale 200 µm; C. Mild inflammation and biliary hyperplasia seen in Striped marsh tadpole, scale 100 µm; D. Higher magnification of *Cystodiscus* spp. (either *C. axonis* or *C. australis*) intra-biliary plasmodium, scale 25 µm; E. Peron's tree frog tadpole with moderate hepatic myxosporidiosis and plasmodia development in gallbladder (free floating forms), scale 200 µm; F. higher magnification view of plasmodia in (E) (40X), plasmodia appear vacuolated and with scattered nuclei in cytoplasm, these are immature forms (presporogonic) of *Cystodiscus* spp. that develop in the gallbladder, scale 40 µm.

The plasmodia of *C. axonis* and *C. australis* develop within the bile ducts as multinucleated intraluminal spherical to tubular structures distributed uniformly across the liver [19], [20]. Plasmodia (Fig. 3) were found in 47% (34/72) of the Green and golden bell frog tadpoles examined (Table 1). Associated lesions consisted of biliary hyperplasia, loss of hepatocytes, and lymphoplasmacytic inflammation at the margins of the expanding lesions (Fig. 3). Biliary hyperplasia was multifocal to focally extensive with lesions coalescing as they became more advanced. The amount of liver affected ranged from less than 10% to in excess of 70%. No evidence of regeneration was noted in any of the sections. Lesions were found in all (n = 34) infected tadpoles. Hepatic lesions were found in 33% (24/72) of tadpoles where plasmodia were not identified.

The presence of hepatic plasmodia was significantly associated with age (tadpole) (p = 0.006) and biliary inflammation (p = <0.001) in univariable logistic regression models (Table S1C). The presence of liver plasmodia, age (tadpole) and season were all significantly associated with the following hepatic lesions within multivariable logistic regression models: biliary hyperplasia (odds ratio = 10.8), loss of hepatocytes (odds ratio = 5.1) and severe hepatic myxosporidiosis (combination of lesions) (odds ratio = 7.9) (Table S1D).

Plasmodia in the gallbladder were observed in 14% (10/74) of Green and golden bell frog tadpoles (confirmed by PCR). Organisms were not attached to the epithelium, and were vacuolated with numerous vegetative nuclei in the cytoplasm, as described by Hartigan *et* *al*. [20]. The immature forms were never observed with myxospores and were distinct from biliary plasmodia based on size and vacuolated appearance. When sectioned, the plasmodia ranged in size from 5–20 μm wide and 50–100 μm long.

### Overwintered Green and golden bell frog tadpoles are significantly more likely to have severe hepatic myxosporidiosis

*Cystodiscus* spp. plasmodia were found in the livers of Green and golden bell frog tadpoles all year round. The prevalence of *Cystodiscus* spp. liver plasmodia in Green and golden bell frog tadpoles was lowest in the spring (22.2%, 2/9), increased in summer (42.9%, 6/8) and winter (44.8%, 13/29). The highest prevalence in the autumn (65.0%, 13/20) (Table 2B), however, the prevalence did not vary significantly between seasons.

Severe hepatic myxosporidiosis was more prevalent in autumn (20.0%, 4/20) and winter (20.1%, 6/29) than spring (0%, 0/9) and summer (14.0%, 2/14) (Table 2C). There was a significant association between the season of collection and the level of disease seen (p = 0.01, Table S1D). Animals collected in winter were 7.9 times more likely to have severe hepatitis than those collected in spring (Table S1D). Severity of hepatic myxosporidiosis was associated with the presence of *Cystodiscus* spp. plasmodia, age and season of collection in a multivariable model (Table S1D).

### Variation in prevalence of hepatic myxosporidiosis in the Striped marsh frog and Peron's tree frog tadpoles

The description of the lesions seen in the Peron's tree frog and Striped marsh frog tadpoles is identical to those described above in Green and golden bell frog tadpoles. Overall chi-sq analysis for liver plasmodia presence in tadpoles was found to be significant (p = 0.0004). Prevalence of Peron's tree frog tadpoles with hepatic plasmodia (86%, 12/14) was significantly higher than seen in the Green and golden bell frog tadpoles (47%, 34/72; χ^2^ p = 0.009) (Table S1E). The majority of infected Peron's tree frog tadpoles (64%, 9/14) had moderate to severe lesions. The prevalence of infection in the Striped marsh frog tadpoles (26%, 10/38) was also significantly lower than in Green and golden bell frog tadpoles (χ^2^ p = 0.033) and only mild lesions were seen in the livers of these tadpoles (Table 1). Early development of gallbladder plasmodia was observed in 36% (5/14) of Peron's tree and 11% (4/38) of Striped marsh frog tadpoles, as described in the Green and golden bell frog tadpoles (Fig. 3).

### *Cystodiscus* infection associated with fibrosis of biliary tracts in adult frogs

**Figure 4:**
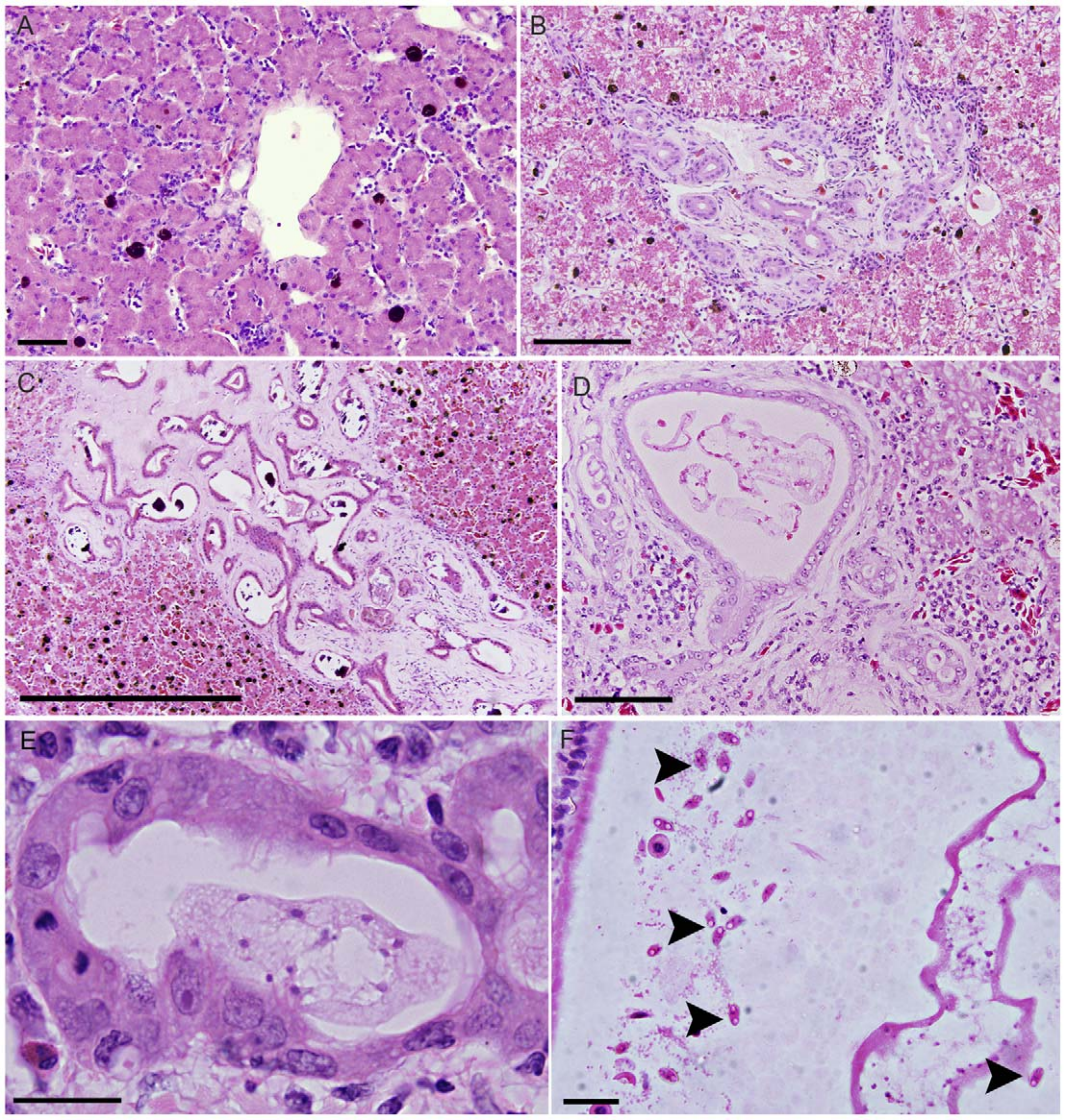
*Cystodiscus* spp. liver and gallbladder development in adult frogs. A. Normal portal triad in healthy adult Striped marsh frog, scale 50 µm; B. Moderate hepatic lesions in Southern bell frog adult with *C. axonis* myxospores in gallbladder, moderate biliary hyperplasia, fibrosis and inflammation. No intra-biliary plasmodia were seen, scale 100 µm; C. Severe biliary fibrosis in adult Peron's tree frog infected with *C. axonis* myxospores in gallbladder. No plasmodia within bile ducts, calcified deposits observed within biliary tree (also seen in tadpoles), scale 500 µm; D. Intrabiliary *C. axonis* plasmodia in sub-adult Yellow spotted bell frog, bile duct extremely distended, surrounded by lymphoplamacytic infiltration and some fibrosis, scale 100 µm; E. *Cystodiscus axonis* plasmodia in adult Booroolong frog bile duct, mild biliary hyperplasia and inflammation accompanied the infection, scale 20 µm; F. Myxospores (arrowheads) in the gallbladder of adult Cane toad, spores identified by circular polar capsules at distal ends, found throughout large plasmodium cytoplasm and freely in bile, scale 40 µm.

Both *C. axonis* and *C. australis* develop spore producing plasmodia in the gallbladder of adult frogs [19], [20]. In histological sections, plasmodia appear as elongated discs containing numerous nuclei and myxospores scattered throughout the cytoplasm and freely in the bile. The oval spores contain characteristic circular polar capsules at distal ends (Fig. 4F).

#### Green and golden bell frogs (*Litoria aurea*)

Twelve Green and golden bell frogs (11 adults and 1 metamorph) were examined in this study for evidence of *Cystodiscus* spp. infection. Biliary plasmodia, biliary hyperplasia and associated loss of hepatocytes, lesions found in tadpole livers, were only found in the metamorph (an animal with 4 legs, but had yet to reabsorb its tail). Myxospores were found in the gallbladder of one adult frog with no biliary lesions were seen.

#### Peron's tree frogs (*Litoria peronii*)

Eight (7 adults and 1 metamorph) Peron's tree frogs were examined. The single metamorph had biliary plasmodia, biliary hyperplasia and associated loss of hepatocytes. Surrounding the proliferating bile ducts within the interstitium were multifocal deposits of amyloid-like eosinophilic fibrillar to amorphous material (Fig. 4C). Immature plasmodia that had not yet begun to produce spores were found in the gallbladder. Six adult frogs (75%, 6/8) had some degree of biliary hyperplasia. This varied from focal to multifocal mild lesions to a moderate locally extensive lesion. At no time did these lesions represent more than 20% of the liver section. Amyloid-like deposits were present in 4 of 5 of the lesions in adult frogs. There was only a moderate deposit of amyloid-like material in 3 frogs but larger deposits were present in the other frog. The prevalence of these lesions (80%, 4/5) was significantly higher than the Green and golden bell frog (0%) (χ^2^ p = 0.002). Five of the frogs (63%, 5/8) with liver lesions had spore producing plasmodia in their gallbladders.

#### Striped marsh frogs (*Limnodynastes peronii*)

Twenty seven adult Striped marsh frog adults were examined and 20 (74%, 20/27) were found to be infected and producing spores. Mild degrees of biliary hyperplasia were found in 12 (60%) of the 20 infected frogs. The prevalence of lesions in the liver (44%, 12/27) was significantly different from those in the Green and golden bell frogs (χ^2^ p = 0.027). The degree of biliary hyperplasia, however, was mild resulting in a two to three fold increase in bile duct cross sections and typically only affected scattered portal triads. A mild degree of peribiliary fibrosis was present in 60% (12/20) of infected frogs, but these changes rarely impacted the surrounding hepatocytes.

#### Booroolong frogs (*Litoria booroolongensis*)

Liver lesions found in the 15 infected adult Booroolong frogs with brain stages included amyloid-like deposits in 20% (3/15), mild periportal fibrosis in 6.7% (1/15) and mild biliary hyperplasia in 20% (3/15). Presporogonic plasmodia were also present in bile ducts of the 3 adult Booroolong frogs (Fig. 4E) with biliary hyperplasia. This is the only time that this stage of development was found in an adult frog.

#### Southern bell frogs (*Litoria raniformis*)

Liver lesions were found in all 8 frogs. These lesions varied from mild biliary hyperplasia with a minimal degree of fibrosis (Fig. 4B), to locally extensive changes with accompanying amyloid-like material and disruption of the adjacent architecture, as seen in the most advanced lesions observed in the Peron's tree frogs. Spore production in the gallbladder was occurring in 7 of 8 frogs.

#### Yellow spotted bell frog (*Litoria castanea*)

Two adults and a sub-adult were examined. The sub-adult had a moderate degree of biliary hyperplasia, with accompanying inflammation and associated loss of adjacent hepatocytes (Fig. 4D). The sub-adult and one adult were found to have an amyloid-like material around the biliary ducts. No frogs were observed to have spores in the gallbladder.

#### Cane toad (*Rhinella marina*)

Six of the nine infected adults examined had fibrosis associated with the portal tract. No biliary hyperplasia or inflammation was observed in any of the livers seen. Large numbers of myxospores were observed in the gallbladders of adults (Fig. 4F).

### Sensitivity of histology to detect *Cystodiscus* species infection in the brain and liver

Throughout this study we used the microscopic examination of single brain and liver histological sections to determine presence or absence of *Cystodiscus* spp. To calculate sensitivity of this approach we compared single histological section outcome with (i) multiple serial histological sectioning and (ii) used a previously developed multiplex PCR outcome [20] on a selection of samples (Table 3).

**Table 3:** Comparison of detection method results (single histological section vs. serial histological sections) and species specific multiplex PCR.

A		
	Serial histological sections	
Single histological section	Positive	Negative
	Tadpole liver samples	
Positive	12	0
Negative	0	5
	Frog and tadpole brain samples	
Positive	3	0
Negative	0	12

B		
	Multiplex PCR	
Single histological section	Positive	Negative
	Tadpole liver samples	
Positive	4	2
Negative	8	5
	Frog and tadpole brain samples	
Positive	3	0
Negative	4	4

N.B Sensitivity for both methods is 100%. Lower 95% confidence Intervals for liver  = 77.9% and for brain  = 36.8%; Upper confidence levels could not be determined.

N.B Sensitivity for liver  = 33.3% (95% CI: 9.9%–65.1%) and for brain  = 42.9% (95% CI: 9.9%–81.6%).

The sensitivity of single vs. serial histological sections for both brain and liver samples was 100%, however within the liver samples, there were four overall positive samples that contained sections in which parasites were not found (false negatives) (5/16; 4/16; 2/30 and 2/34). Importantly, observing all serial sections including the false negative sections revealed histopathological lesions characteristic of hepatic myxosporidiosis (biliary hyperplasia, inflammation or loss of hepatocytes).

*Cystodiscus* was detected histologically in 43% of brains, and 33% of livers which tested positive by multiplex PCR. PCR was 100% sensitive in detecting brains that were microscopically *Cystodiscus* was observed, but since it did not detect infection in 2/19 liver samples that were positive on histology, sensitivity of PCR in liver is 66% (Table 3).

The limit of detection using 10-fold serial dilutions of *C. axonis* and *C. australis* myxospores DNA (Figure S1) was equivalent to 1 myxospore, as confirmed by three independent PCR reactions. An equivalent of 0.1 myxospore DNA did not return species specific PCR amplicon for either *Cystodiscus* spp.

## Discussion

### Ecological implications of *C. axonis* and *C. australis* for Australian frogs

*Cystodiscus axonis* and *C. australis* are emerging myxosporean parasites of multiple species of Australian frogs. In addition to characterizing the prevalence and pathology associated with *C. axonis* and *C. australis* infection in several amphibian populations, the question that we address in this paper is whether these parasites have an ecological impact. Several biological characteristics are necessary for an emerging pathogen to have a significant and persistent ecological impact. The pathogen must be able to infect a high percentage of the susceptible population, it must be able to cause disease in a significant number of the animals that it infects, the disease that it generates must be sufficiently severe that it will directly or indirectly interfere with recruitment of juvenile animals into the breeding population or kill mature breeding adults, and the pathogen needs to persist in the environment [31]. In this study, we show that *C. axonis* and *C. australis* exhibit these characteristics in our study populations and thus have a significant potential for ecosystem impact.

Our findings show that *C. axonis* and *C. australis* caused high prevalences of infection in multiple species of frogs including the endangered Green and golden bell frog where prevalences of infection in tadpoles were as high as 65%. They also produced significant disease that directly or indirectly would have impacted their host in the common and endangered species of frog examined in this study. Neurological dysfunction is associated with similar haemorrhagic lesions in fish infected with myxosporea in the brain (*Myxobolus balantiochelois* and *Myxobolus cerebralis*) [32]–[34]. The inflammatory and haemorrhagic lesions associated with *C. axonis* described here and possibly the presence of the parasite within axons would be expected to significantly interfere with neurological function and this was indeed the case in some frogs that were observed prior to death. The ecological impact of the nervous system form of *C. axonis* may be increased by its ability to cause disease in both tadpoles and adult frogs. Significant brain lesions were seen in a high percentage in adults of both the Southern bell frog and the Yellow spotted bell frog. However, whether these types of lesions occur in the wild is unknown. In this study, all the adult frogs with *C. axonis* lesions in the nervous system where held in captivity for months to years before their death. The stress of captivity can exacerbate the impacts of pathogens and disease [35], and it is possible that the stress or advanced age (in some cases) could have made these frogs more prone to disease.

Both *C. axonis* and *C. australis* were associated with severe disease in the liver of tadpoles of susceptible species. A healthy tadpole liver is essential for the hormonal cascades that bring about metamorphosis [36], in addition the liver is the site for haematopoesis, immunoglobulin production [37], and is an important organ for energy and protein metabolism [38]. It is likely that the extent of the lesions seen in the livers of the Green and golden bell frog tadpoles and the Peron's tree frog tadpoles would have significant metabolic and physiological implications. Our seasonal data also suggests that the liver disease resulted in a delayed metamorphosis and overwintering in some Green and golden bell frog tadpoles.

Overwintering usually occurs when tadpoles do not have the resources to metamorphose and provides the advantage of extra time to feed and grow before spring [39]. However, overwintering also increases the risk of desiccation [40], predation and increased exposure to pathogens and parasites [39]. It is unclear if infection with *Cystodiscus* spp. predisposes tadpoles to overwinter or if overwintered tadpoles are just infected for longer and therefore have a severe (chronic) host response. Regardless, any negative impact on the ability to metamorphose exposes tadpoles to more risks and removes one reproductive season from the life history of the tadpole/frog.

The ability of *C. axonis* and *C. australis* to have spread so widely in 50 years could suggest an ability to persist in the environment. This could be explained by the resilience of myxospores in the water column as described for other myxosporea species [41], or sustained infection in other (vertebrate and invertebrate) hosts. Subclinical but productive infections were observed in the invasive Cane toad and at least one species of widely distributed native frog, the Striped marsh frog [19]. Neither of these species developed significant disease as either tadpoles or adults, but both species produced myxospores in their gallbladders and would have been expected to shed the parasites during their adult life. Similar differences in susceptibility of native species to an invasive pathogen was been shown in studies of the chytrid fungus (*Batrachochytrium dendrobatidis*), where Striped marsh frogs were shown to have a higher tolerance for infection than the Green and golden bell frogs [42].

### Is presporogonic plasmodial development in the nervous system an amplification strategy?

Presporogonic development was observed to be a common finding in the nervous system of tadpoles and adult frogs infected with *C. axonis*. The question then arises as to whether it is a necessary part of the parasites life cycle? Based on the myxosporea of fish, the nervous system can act as a route of infection and conduit to other tissues, as occurs with *M. cerebralis* in salmonid fish [43], or as site of spore production with spores being released when the host dies or is eaten as occurs in several other species of fish [9], [10], [44]–[46]. Evidence that the nervous system is used by *C. axonis* for amplification is based on the finding that the nervous system was infected by *C. axonis* in all species of frogs examined, arguing that it was a targeted tissue. Additionally, finding the organisms in cranial and peripheral nerves suggests that *C. axonis* may use the nervous system as an entry way to the tadpole. Lastly, finding organisms in close association with vessels could explain how the parasite moved from the central nervous system to other tissues as has been suggested for other myxosporea species [47], [48]. If *C. axonis* does use the nervous system as a site for amplification within the host it is likely that in its natural host it causes minimal or no disease as was seen in the Cane toad and the Striped marsh frog. Adverse impacts, as seen in Green and golden bell frog tadpoles and Booroolong frogs, and adults of the Southern bell frog and Yellow spotted bell frogs appear to represent reaction of the host's immune system to the parasite, and would be those expected in non-parasite adapted species. Ultimately, to determine if the nervous system stages of *C. axonis* represent an important developmental stage controlled infection trials where the sequence of the development of *C. axonis* can be followed will be necessary.

### Possible window of vulnerability and management implications

From a conservation perspective, it would be valuable to know if *Cystodiscus* infections occur only in the tadpole or at any time during the tadpole and frog life cycles. Based on studies of myxosporean infections in fish, age of the host and seasonal factors would be expected to impact susceptibility to infection and disease [49]–[51]. Tadpoles have also been shown to exhibit different levels of disease and resistance to other parasites, for example the trematode (Ribeiroia ondatrae). In this case the type and severity of limb malformations varied according to tadpole their size and developmental stage [52].

The difference in prevalence between frog and tadpoles for both *Cystodiscus* species raises questions about the timing of infection. The low rate of infection in adult Green and golden bell frogs, compared to tadpoles suggests that infected tadpoles are either capable of ridding themselves of infection, or infected tadpoles have reduced survival rates compared with non-infected animals, or that adults of this species may be resistant to infection. Whether this represents infection in adult animals or infection of tadpoles with delayed development will require controlled experiments to determine. If *Cystodiscus* spp. are only able to infect tadpoles or only tadpoles of specific ages this could be valuable information for establishing captive bred populations that are free of disease and strategies for translocation of tadpoles.

The presence of presporogonic plasmodia in a small number of adult Booroolong frogs infected with *C. axonis* was unexpected because these stages were never seen in adult frogs of any other species in this study. These animals were euthanized immediately after capture from the wild and thus could have been recently infected. Alternately, the liver could have been colonized by reactivation of a latent infection in the brain or in other tissue. Why the bile duct environment could support presporogonic plasmodia in this species and not in others remains unknown and would require controlled infection trials to determine.

### PCR and histology are valuable surveillance tools for *Cystodiscus* infections in frogs

Screening frogs and tadpoles for infectious diseases is necessary to prevent the introduction of disease into captive breeding programs, preventing disease release into new areas from captive-raised frogs, and determining the geographic distribution of the disease. This study shows that both PCR and histopathology have advantages as screening tools for *Cystodiscus* infections and that a combination of both is optimum.

The PCR assay used in this study amplified an rDNA sequence for which as many as 10,000 copies may be present in mxyosporeans [53]. It was found to be very sensitive (sensitivity limit one organism) and was more sensitive than histology in detecting infection. This is consistent with a similar study, where PCR was found to be up to 3 times more sensitive than histology for detecting infection with the *B. dendrobatidis* [54]. Our PCR was also able to detect and distinguish between infection with one or both species of *Cystodiscus*, and the added advantage of PCR is that it can be used with pooled samples. The only disadvantage of PCR would be is susceptibility to contamination. Proving that animals were actually infected and that samples were not contaminated by environmental organisms can be difficult as has been shown for other amphibian pathogens in aquatic environments [54].

Histology as a screening tool has the advantages that it can be used to detect an array of infectious agents, in addition to *Cystodiscus* spp., and that it can determine if infection is associated with disease. Combining these screening techniques or using histopathology as a confirmatory tool for populations that have been shown to be PCR positive would provide the most diagnostic power. Whichever technique is used, screening tadpoles in summer or autumn is optimal to detect infected populations as this is when infection prevalence is highest. In addition, development of an *in situ* hybridisation protocol for these two species could prove extremely informative for future research in these parasites.

This study shows that pathologists should consider *C. axonis* in Australian frogs (tadpoles and adults) if they are exhibiting gliosis and infection with either *C. axonis* or *C. australis* or both in tadpoles with biliary hyperplasia, particularly if there is minimal associated inflammation. Differential diagnoses for the lesions caused by *C. axonis* in the nervous system would be those caused by the rare extension of systemic bacterial and fungal infections to the nervous system and these would typically be accompanied by a neutrophilic or granulomatous inflammation. Liver disease caused by infectious agents (viruses, bacteria, other parasites and fungi), however, is common in frogs and tadpoles [55]–[58]. These diseases differ from the lesions caused by *Cystodiscus* spp. by the level of necrosis observed, multiple organs affected and the physical presence of the pathogen, e.g. fungal hyphae or nematode larvae.

### Conclusion

This study shows that both *Cystodiscus axonis* and *Cystodiscus australis* have the potential to infect and cause disease in common and endangered Australian frog species. They were also associated with spontaneous deaths in semi captive and captive populations of frogs. If these infection dynamics occur in wild frogs then they would also be expected to significantly reduce tadpole recruitment and adult frog longevity, potentially making them a key threatening process. Additionally, this study also provides evidence of certain host developmental stages being more susceptible to infection, which may have significant implications for release of captive bred animals. Lastly, this study provides investigators the necessary tools for screening wild and captive populations of frogs for the presence of these parasites and these tools may prove instrumental in determining the distribution of these parasites in Australia and establishing specific pathogen free captive breeding colonies.

## Supporting Information

Figure S1 **Detection limit for multiplex species specific PCR.** Ten fold dilutions of quantified myxospore samples for *Cystodiscus axonis* and *C. australis* using *Cystodiscus* internal transcribed spacer rDNA specific primers. Reactions were run in triplicate and visualised on a 2% agarose gel stained with Gel Red (Biotium, Australia).
(TIF)

Table S1 Summary of statistical analyses results for associating *Cystodiscus axonis* and *Cystodiscus australis* with lesions of disease.
(DOC)
