# Supplementary figures and images for: Comparative Pathology and Ecological Implications of Two Myxosporean Parasites in Native Australian Frogs and the Invasive Cane Toad
Source: PLoS One. 2012 Oct 3;7(10):e43780. doi: 10.1371/journal.pone.0043780 (PMC3463585; doi:10.1371/journal.pone.0043780)

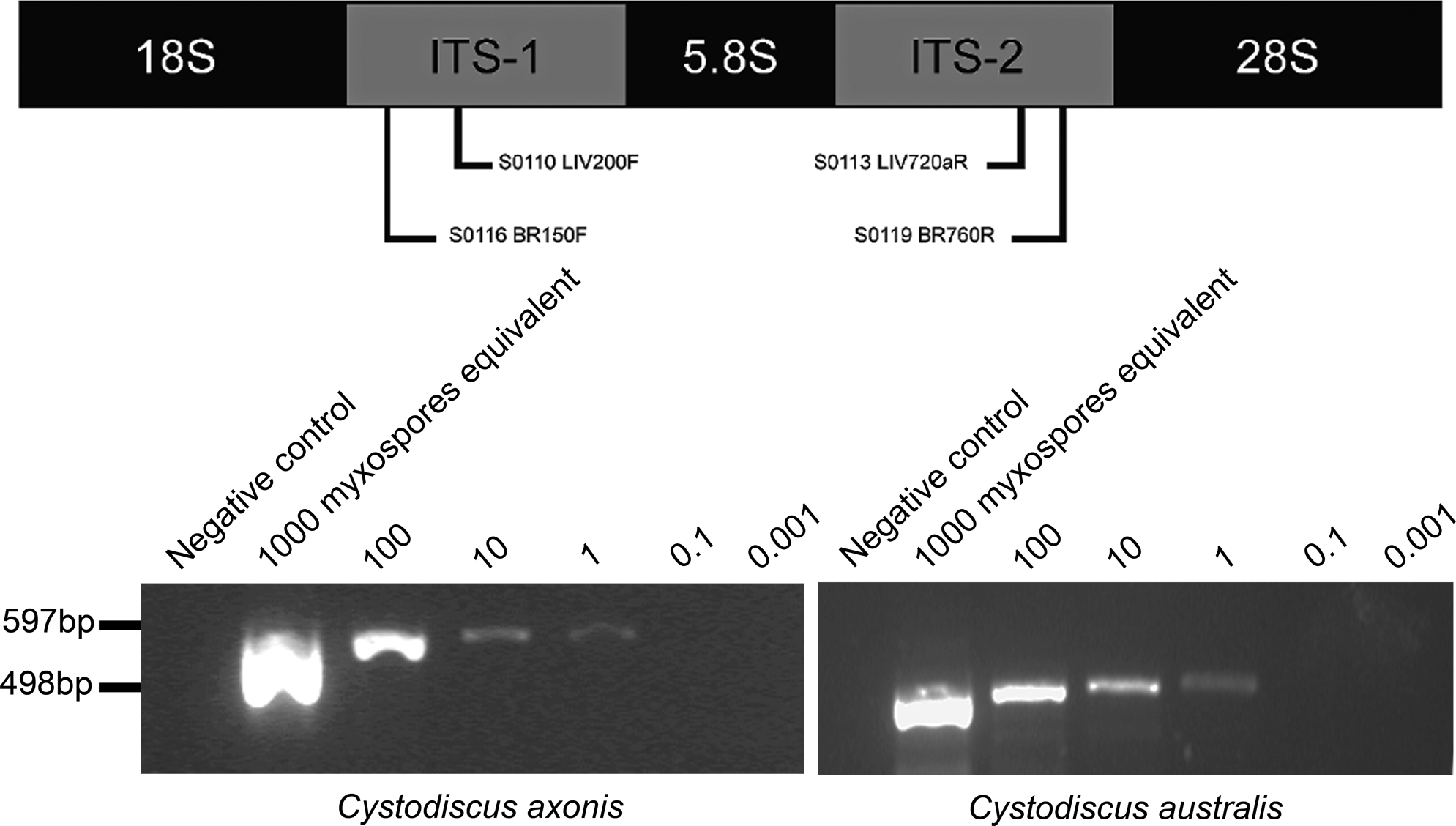

Supplement: Figure S1 — Detection limit for multiplex species specific PCR. Ten fold dilutions of quantified myxospore samples for Cystodiscus axonis and C. australis using Cystodiscus internal transcribed spacer rDNA specific primers. Reactions were run in triplicate and visualised on a 2% agarose gel stained with Gel Red (Biotium, Australia). (TIF) [file pone.0043780.s001.tif]
