# Supplementary material for: Comparative Pathology and Ecological Implications of Two Myxosporean Parasites in Native Australian Frogs and the Invasive Cane Toad
Source: PLoS One. 2012 Oct 3;7(10):e43780. doi: 10.1371/journal.pone.0043780 (PMC3463585; doi:10.1371/journal.pone.0043780)
Supplement: Table S1 — Summary of statistical analyses results for associating Cystodiscus axonis and Cystodiscus australis with lesions of disease. (DOC) [file pone.0043780.s002.doc]

**Table 2A-E. Summary of statistical analyses results for associating *Cystodiscus axonis* and *Cystodiscus australis* with lesions of disease**

| **A** | | | | | | |  | |  |  |  |  |
| --- | --- | --- | --- | --- | --- | --- | --- | --- | --- | --- | --- | --- |
| **Brain histopathology (GGBF)** | |  | | | | |  | | | | | |
| **Univariable binary logistic regression** |  |  |  |  |  |  | |  | | | | |
| **Outcome** | **Explanatory variables** | **b** | **s.e.** | **Odds Ratio** | **95% C.I** | **p** | |  | | | | |
| Presence of brain plasmodia | Age (Adult versus Tadpoles) | 1.04 | 0.79 | 2.83 | -0.64, 2.57 | 0.21 | |  | | | | |
|  | Season |  |  |  |  | 0.003* | |  | | | | |
| Gliosis | Presence of brain plasmodia** | 3.75 | 0.89 | 42.50 | 8.83, 325.64 | <0.001 | |  | | | | |
|  | Parasitic load |  |  |  |  | <0.001* | |  | | | | |
| *Fisher’s exact test   | **B** |  |  |  |  |  |  | | --- | --- | --- | --- | --- | --- | --- | | **Chi-sq test** |  |  |  |  |  | **p** | | Presence of brain plasmodia | Striped marsh frog tadpoles versus GGBF |  |  |  |  | 0.0086 | | Peron's tree frog tadpoles versus GGBF |  |  |  |  | 0.0004 | | | | | | | |  | |  |  |  |  |

| **C** | |  |  |  |  |  |  |
| --- | --- | --- | --- | --- | --- | --- | --- |
| **Liver histopathology (GGBF)** | | |  |  |  |  |  |
| **Univariable binary logistic regression** |  | |  |  |  |  |  |
| **Outcomes** | **Explanatory variables** | | **b** | **s.e.** | **Odds Ratio** | **95% C.I** | **p** |
| Presence of Liver plasmodia | Age (Adult versus Tadpoles) | | -2.29 | 1.07 | 0.10 | 0.005, 0.564 | 0.006 |
| Biliary inflammation | Presence of liver plasmodia | | 2.245 | 0.481 | 9.438 | 3.78, 25.32 | <.0001 |

| **D** |  |  |  |  |  |  |
| --- | --- | --- | --- | --- | --- | --- |
| **Multivariable logistic regression** |  |  |  |  |  |  |
| **Outcomes** | **Explanatory variables** | **b** | **s.e.** | **Odds Ratio** | **95% C.I** | **p** |
| Biliary hyperplasia | Intercept | -0.8315 | 0.7414 |  |  |  |
|  | Presence of liver plasmodia | 2.5827 | 0.8454 | 13.233 | 3.0, 95.4 | 0.0023 |
|  | Age (Adults versus Tadpoles) | -2.2683 | 1.0627 | 0.103 | 0.009, 0.68 | 0.0328 |
|  | Season |  |  |  |  | 0.0285 |
|  | Summer vs. Spring | 0.167 | 0.9708 | 1.182 | 0.17, 8.38 | 0.86 |
|  | Autumn vs. Spring | 1.5394 | 0.9679 | 4.662 | 0.76, 36.42 | 0.11 |
|  | Winter vs. Spring | 2.3841 | 0.968 | 10.849 | 1.79, 85.49 | 0.01 |
| Grade of biliary hyperplasia | Intercept 3 | -4.48 | 0.87 |  |  |  |
|  | Intercept 2 | -1.85 | 0.71 |  |  |  |
|  | Intercept 1 | -0.76 | 0.68 |  |  |  |
|  | Presence of liver plasmodia | 2.87 | 0.58 | 17.70 | 5.98, 61.83 | <0.001 |
|  | Age (Adults versus Tadpoles) | -2.25 | 0.92 | 0.11 | 0.01, 0.56 | 0.015 |
|  | Season |  |  |  |  | 0.057 |
|  | Summer vs. Spring | 0.43 | 0.84 | 1.53 | 0.28, 9.40 | 0.61 |
|  | Autumn vs. Spring | 1.60 | 0.80 | 4.96 | 1.05, 28.20 | 0.044 |
|  | Winter vs. Spring | 1.62 | 0.76 | 5.06 | 1.16, 26.67 | 0.034 |
| Loss of hepatocytes | Intercept | -1.95 | 0.92 |  |  |  |
|  | Presence of liver plasmodia | 2.12 | 0.68 | 8.33 | 2.39, 36.37 | 0.002 |
|  | Age (Adults versus Tadpoles) | -2.69 | 1.28 | 0.07 | 0.003, 0.61 | 0.036 |
|  | Season |  |  |  |  | 0.002 |
|  | Summer vs. Spring | -0.58 | 1.15 | 0.56 | 0.06, 6.05 | 0.62 |
|  | Autumn vs. Spring | 2.28 | 1.06 | 9.75 | 1.41, 100.2 | 0.032 |
|  | Winter vs. Spring | 2.43 | 1.00 | 11.35 | 1.86, 106.76 | 0.016 |
| Hepatic myxosporidiosis severity | Intercept 3 | -4.59 | 0.84 |  |  |  |
|  | Intercept 2 | -2.90 | 0.75 |  |  |  |
|  | Intercept 1 | -0.73 | 0.67 |  |  |  |
|  | Presence of liver plasmodia | 2.42 | 0.52 | 11.22 | 4.21, 32.41 | <0.001 |
|  | Age (Adults versus Tadpoles) | -2.04 | 0.94 | 0.13 | 0.01, 0.695 | 0.03 |
|  | Season |  |  |  |  | 0.01 |
|  | Summer vs. Spring | 0.46 | 0.83 | 1.58 | 0.30, 8.99 | 0.58 |
|  | Autumn vs. Spring | 1.08 | 0.78 | 2.95 | 0.67, 15.23 | 0.16 |
|  | Winter vs. Spring | 2.07 | 0.77 | 7.91 | 1.88, 36.65 | 0.007 |

| **E** |  |  |  |  |  |  |
| --- | --- | --- | --- | --- | --- | --- |
| **Chi-sq test** |  |  |  |  |  | **p** |
| Liver plasmodia | GGBF vs. Striped marsh frog tadpoles |  |  |  |  | 0.03331 |
|  | GGBF vs. Peron's tree frog tadpoles |  |  |  |  | 0.00824 |
| Biliary fibrosis | GGBF vs. Striped marsh frog adults |  |  |  |  | 0.02725 |
|  | GGBF vs. Peron's tree frog adults |  |  |  |  | 0.0022 |
